# Vaccination Coverage and Awareness on Hepatitis B Virus Among High School, College, and University Students of Rajshahi Division of Bangladesh: A Cross‐Sectional Study

**DOI:** 10.1002/hsr2.71871

**Published:** 2026-02-20

**Authors:** Mst. Hajera Khatun, Md. Arif Hossain, Jaytirmoy Barmon, Mst. Sharmin Khatun, Md. Milton Hossain, Md. Minhazul Islam, Al Mamun, Md. Ajijur Rahman

**Affiliations:** ^1^ Department of Pharmacy, School of Science and Engineering Varendra University Rajshahi Bangladesh; ^2^ BCSIR Rajshahi Laboratories, Oils, Fats and Waxes Research Division, Bangladesh Council of Scientific and Industrial Research (BCSIR) Rajshahi Bangladesh; ^3^ Department of Pharmacy Faculty of Science University of Rajshahi Rajshahi Bangladesh

**Keywords:** attitudes, Bangladesh, hepatitis B, knowledge, vaccination

## Abstract

**Background:**

Hepatitis B virus (HBV) causes hepatitis B, a vaccine‐preventable liver disease. The purpose of the present study was to investigate the knowledge and attitudes toward HBV and its vaccination among students of the Rajshahi Division of Bangladesh.

**Methods:**

In this cross‐sectional survey, data were collected by filling out a well‐structured questionnaire. SPSS (IBM version 23), chi‐square (*χ* ^2^) tests, and a binary logistic regression model were used for the data analysis.

**Results:**

A binary logistic regression model showed that a significantly lower rate of vaccination status was observed among participants from the university (44.44%) than those from college (42.11%) and high school (86.3%) [OR = 1.917, 95% CI = 0.752–17.869, *p* = 0.001]. A higher proportion of male students (58.88%) remained unvaccinated compared to female students (43.1%) [OR = 1.435, 95% CI = 1.253–1.873, *p* = 0.027]. Regarding the knowledge of HBV and its vaccination, university students had better knowledge than high school students (*p* > 0.05).

**Conclusion:**

The results of the study clearly highlight the need to increase awareness among this group of people about HBV and its vaccination to eradicate its infection from the community.

**Abbreviations**
CI: confidence interval
HBV: hepatitis B virus
OR: odds ratio

## 1 Background

Hepatitis B virus (HBV), a member of the Hepadnaviridae family, is a tiny DNA virus with bizarre characteristics resembling retroviruses [1]. The genetic material of the virus is made of DNA [1]. Primary liver cells or hepatocytes are productively infected with HBV, which is replicated by reverse transcription [2, 3]. After infection, the circular and partially double‐stranded DNA of the HBV virion is transformed into covalently closed circular DNA (cccDNA) in the nucleus, creating a minichromosome that acts as a template for the production of viral mRNA [4]. A liver infection can be either short‐term (6 months) or chronic (lifelong), depending on how well the host immune system fights off the infection. Immune‐mediated liver damage, which includes hepatocellular carcinoma (HCC) and liver cirrhosis (LC) [5], poses a significant public health threat worldwide due to HBV infection. HBV infections are delayed but not cured by antiviral therapies using nucleoside analog inhibitors of viral DNA production because cccDNA remains persistent in hepatocytes [6].

Given the biological characteristics of HBV, it is no surprise that HBV infection remains a major public health concern worldwide. Globally, an estimated 240 million people are chronically infected with HBV, resulting in ~686,000 deaths per year owing to HBV consequences such as LC and HCC [7]. Bangladesh has a high prevalence of HBV infection. In Bangladesh, ~3–8 million people have a chronic HBV infection [8] and about 1 million of these people will eventually develop acute‐or‐chronic liver failure (ACLF), LC, or HCC [8]. Moreover, in Bangladesh, HBV‐related liver illnesses are thought to be the primary cause of hospital admissions and cause ~20,000 fatalities annually, despite the lack of accurate information [8].

The virus spreads through percutaneous or perimucosal contact with contaminated blood or bodily fluids and has a 40–160 day incubation period (average 60–90 days) [9]. Its transmission can occur by vertical transmission from an infected mother to her child, horizontal transmission (e.g., within a home), sexual transmission, or parenteral transmission (e.g., via injecting drugs, sharps injuries, or contaminated blood products) [10].

HBV infection can be prevented using an easily accessible vaccination regimen [11]. The HBV vaccine was the first anticancer vaccine with documented safety and efficacy records. It has a 95% success rate in preventing chronic infections in both adults and children [12]. More importantly, the prevalence of HBV infection has declined globally with the introduction of HBV vaccine and other preventive measures [11].

In 2005, Bangladesh implemented the HBV vaccine as part of an expanded program on immunization (EPI), adhering to the WHO's recommended vaccination schedules at 6, 10, and 14 weeks of age [13]. Various socioeconomic factors have been shown to affect the childhood immunization rates in Bangladesh [14]. Mothers living in the lowest wealth quintile and having less education have a lower chance of having fully vaccinated children because they visit vaccination care units less frequently [15]. Consequently, complete immunization coverage in Bangladesh remains elusive. Furthermore, a low vaccination rate has been demonstrated among medical students of Bangladesh [16]. The stigma surrounding infectious diseases can affect public attitudes. In some cases, misconceptions and lack of understanding of transmission may contribute to discrimination. Preventing the spread of this virus requires knowledge of the channels of transmission, risks associated with treatment, and the use of proper immunization. Furthermore, people may be reluctant to disclose their HBV status or get tested because of fear of social rejection or prejudice. In addition, they may avoid healthcare providers to keep their status secret. This can lead to undetected cases, increasing the risk of unknowingly spreading the virus. However, low vaccination rates, particularly in high‐risk populations, might be caused by misinformation or cultural attitudes that undermine trust in health authorities or vaccinations. Students represent a key demographic for preventative health interventions, as they are often at increased risk for exposure to infectious diseases and play a vital role in disseminating health information within their communities. Therefore, the present study was designed to evaluate the level of knowledge, attitudes, and vaccination status regarding HBV among students aged 14–30 in the Rajshahi Division of Bangladesh.

## 2 Methods

### 2.1 Study Area

The survey was conducted at several high schools, colleges, and universities located in the Rajshahi Division of Bangladesh. Rajshahi is one of eight first‐level administrative divisions in Bangladesh. We conducted our study in six of the eight districts of the Rajshahi Division: Rajshahi, Chapainawabganj, Naogaon, Bogura, Sirajganj, and Pabna owing to accessibility.

### 2.2 Study Period

Data were collected between July 2021 and October 2021.

### 2.3 Study Population

In this study, we selected high school, college, and university students aged 14–30 years. A total of 700 students from different institutions in the Rajshahi Division were selected for data collection using random sampling. A randomly selected sample was intended to be an objective depiction of the entire population. An objective random sample is essential to draw conclusions.

### 2.4 Data Collection Techniques

**Figure 1:**
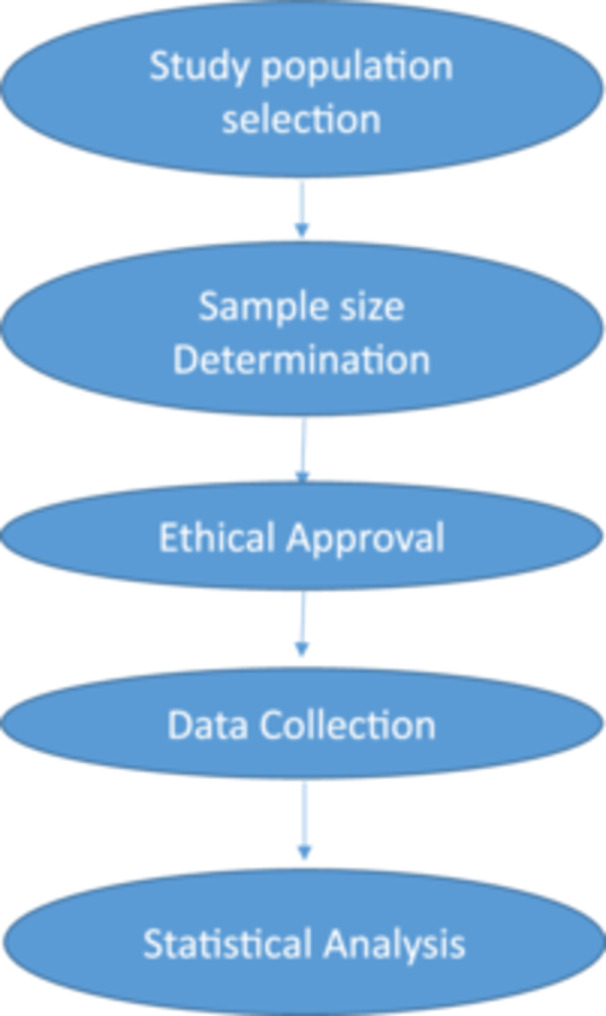
Flow chart of methodology.

First, we informed participants about the contents of the consent form. Before collecting data, we provided information sheets and consent forms to the participants. Written informed consent was obtained from the study participants for the use of anonymous personal data in research. Confidentiality of the information was maintained thoroughly by de‐identification. We collected data from the participants who provided their consent. We then collected the data by directly filling out the questionnaire by the participants. Therefore, we accepted the social desirability, recall, misunderstanding, response, and nonresponse biases. All the questionnaires were translated into Bengali for easy understanding to give the answer. The overall methodology of this study is shown in Figure 1.

### 2.5 Independent Variable

Sociodemographic, HBV and vaccination‐related knowledge and attitudes were considered as independent variables in this study [17, 18]. These independent variables were selected on the basis of previous studies. The independent variables with groups are given in Table 1.

**Table 1:** List of independent variables with group.

Variable	Group	Variable	Group
Gender	Male	Is HBV curable?	Yes
	Female		No
		Does HBV cause liver cancer?	Yes
Marital status	Married		No
	Unmarried	Can HBV lead to death?	Yes
Educational status	High school		No
	College	Do you feel uncomfortable sitting with a HBV‐infected person?	Yes
	University		No
Do you know about HBV?	Yes	You do not mind shaking hands/hugging with a HBV‐infected person.	Yes
	No		No
HBV is a type of…	Virus	Do you know that HBV can be prevented by vaccination?	Yes
	Bacteria		No
	Fungi	Do you help your family member to take vaccine against HBV?	Yes
	Parasites		No
	None		
Is HBV sexually transmitted disease?	Yes		
	No	Do you suggest your friends, relatives, or other persons to take vaccine against HBV?	Yes
Is HBV transmitted by kissing?	Yes		No
	No		

### 2.6 Outcome Variable

The outcome variable of this study was vaccination status among the study population. The samples were classified as (i) vaccinated or (ii) not vaccinated.

### 2.7 Statistical Analysis

Statistical analysis was performed using SPSS (IBM version 23). Descriptive statistics such as frequencies and percentages (for qualitative variables) and mean ± standard deviation (for continuous variables) were calculated. Furthermore, *χ* ^2^ tests, along with a binary multivariate logistic regression model, were used to identify the predictive factors related to vaccination status. Multivariate logistic regression adjusted for sociodemographic factors and vaccination status. A *p* significance was set at *p* < 0.05. However, a small *p* value indicates a statistically significant result but says nothing about how large or meaningful the effect is. In our logistic regression models, an OR > 1 indicates higher odds of being unvaccinated compared to the reference group, unless otherwise specified. No missing data were identified in this study, as confirmed through analysis using SPSS software.

## 3 Results

### 3.1 Demographic Characteristics of the Participants

The distribution of the participants' sociodemographic traits is shown in Table 2. Of the total sample (*n* = 700), 42.57% (*n* = 298/700) were male and 57.43% (*n* = 402/700) were female. The majority of participants were university students (58%, *n* = 406/700), followed by high school students (31%, *n* = 217/700) and college students (11%, *n* = 77/700). In total, 94% (*n* = 658/700) of respondents were unmarried.

**Table 2:** Demographic characteristics of the study sample.

Variables	Frequency	Percentage
Gender		
Male	298	42.57
Female	402	57.43
Marital status		
Married	42	6
Unmarried	658	94
Educational status		
High school	217	31
College	77	11
University	406	58

### 3.2 Assessment of Knowledge‐Related Characteristics About HBV and Its Vaccination With Sociodemographic Variables

College (100%) and university (99.5%) students demonstrated significantly higher awareness that HBV is a virus compared to high school students (95.43%), with a *p* value of 0.031 (< 0.05). Additionally, this knowledge was more prevalent among unmarried students (98.48%) than married students (95.35%), with a statistically significant difference (*p* = 0.001). However, no significant association was observed based on gender (*p* > 0.05).

The study found that participants' knowledge about the mode of transmission was significantly influenced by their gender and educational status, while marital status had no apparent effect (Table 3). Specifically, 56.79% of university students, 39.47% of college students, and 36.53% of high school students correctly identified HBV as a sexually transmitted disease.

**Table 3:** Sociodemographic and knowledge‐related characteristics about HBV and its vaccination of the study sample.

Characteristics	Gender			Marital status			Educational status			
	Male *n* (%)	Female *n* (%)	*p*	Married *n* (%)	Unmarried *n* (%)	*p*	High school *n* (%)	College *n* (%)	University *n* (%)	*p*
Do you know about HBV?	Total (*n* = 700)									
Yes	272 (91.58)	388 (96.28)	**0.013**	40 (93.02)	620 (94.37)	0.730	211 (96.35)	72 (94.74)	394 (97.28)	**0.042**
No	25 (8.42)	15 (3.72)		3 (6.98)	37 (5.63)		8 (3.65)	4 (5.26)	11 (2.72)	
HBV is a type of…	Total (*n* = 700)									
Virus	289 (97.31)	399 (99.01)	0.062	41 (95.35)	647 (98.48)	**0.001**	209 (95.43)	76 (100)	403 (99.5)	**0.031**
Bacteria	3 (1.01)	1 (0.25)		0	4 (0.61)		4 (1.83)	0	0	
Fungus	2 (0.67)	3 (0.74)		0	5 (0.76)		5 (2.28)	0	0	
Parasites	1 (0.34)	0		0	1 (0.15)		1 (0.46)	0	0	
None	2 (0.67)	0		2 (4.65)	0		0	0	2 (0.5)	
Is HBV sexually transmitted disease?	Total (*n* = 700)									
Yes	142 (47.81)	257 (63.77)	**< 0.001**	27 (62.79)	372 (56.62)	0.525	80 (36.53)	30 (39.47)	230 (56.79)	**0.001**
No	155 (52.19)	146 (36.23)		16 (37.21)	285 (43.38)		139 (63.47)	46 (60.53)	175 (43.21)	
Is HBV transmitted by kissing?	Total (*n* = 699)									
Yes	77 (25.93)	72 (17.91)	**0.012**	10 (23.26)	139 (21.19)	0.704	110 (50.46)	18 (23.68)	97 (23.95)	**0.018**
No	220 (74.07)	330 (82.09)		33 (76.74)	517 (78.81)		184 (49.54)	58 (76.32)	308 (76.05)	
Is HBV curable?	Total (*n* = 700)									
Yes	243 (81.82)	360 (89.33)	**0.006**	35 (81.4)	568 (86.45)	0.361	201 (91.78)	54 (71.05)	348 (85.93)	0.111
No	54 (18.18)	43 (10.67)		8 (18.6)	89 (13.55)		18 (8.22)	22 (28.95)	57 (14.07)	
Does HBV cause liver cancer?	Total (*n* = 700)									
Yes	251 (84.51)	357 (88.59)	0.141	37 (86.04)	571 (86.91)	0.817	154 (70.32)	55 (72.37)	358 (88.4)	**0.002**
No	46 (15.49)	46 (11.41)		6 (13.96)	86 (13.09)		65 (29.68)	21 (27.63)	47 (11.6)	
Can HBV lead to death?	Total (*n* = 698)									
Yes	276 (93.56)	354 (87.84)	**0.014**	39 (90.7)	591 (90.23)	1.000	185 (84.47)	66 (89.19)	359 (88.64)	0.051
No	19 (6.44)	49 (12.16)		4 (9.3)	64 (9.77)		34 (15.53)	8 (10.81)	46 (11.36)	
Do you know that HBV can be prevented by vaccination?	Total (*n* = 700)									
Yes	264 (88.89)	378 (93.8)	**0.026**	42 (97.67)	600 (91.32)	**0.046**	199 (90.87)	72 (94.74)	384 (94.81)	0.831
No	33 (11.11)	25 (6.2)		1 (2.33)	57 (8.68)		20 (9.13)	4 (5.26)	21 (5.19)	

*Note:* Bold values indicate statistically significant.

Female students (63.77%) were also significantly more likely to recognize HBV as a sexually transmitted disease compared to male students (*p* = 0.001). Additionally, 23.95% of university students believed that HBV can be transmitted through kissing, compared to 50.46% of high school students and 23.68% of college students. Notably, a significantly higher proportion of male students (25.93%) held this belief compared to female students (17.91%) (*p* = 0.012).

According to our findings, 89.33% (*n* = 360/403) of female students and 81.82% (*n* = 243/297) of male students believed that hepatitis B is curable. A significant difference in responses was also observed among university, high school, and college students when they were asked whether HBV causes liver cancer (*p* = 0.002). Specifically, 88.4% (*n* = 358/405) of university students reported that HBV causes liver cancer, compared to 70.32% (*n* = 154/219) of high school students and 72.37% (*n* = 55/76) of college students.

Furthermore, 93.56% (*n* = 276/295) of male students significantly agreed that HBV infection can lead to death, compared to female students (*p* = 0.014). However, no statistically significant difference was found in the understanding of prevention across high school, college, and university students, with most agreeing that vaccination can prevent hepatitis B.

In contrast, a significantly higher proportion of female (93.8%, *n* = 378/403) and married (97.67%, *n* = 42/43) students demonstrated knowledge about HBV prevention compared to male (88.89%, *n* = 264/297) and unmarried (91.32%, *n* = 600/657) students (*p* = 0.026 and *p* = 0.046, respectively).

### 3.3 Assessment of Attitude‐Related Characteristics About HBV and Its Vaccination With Sociodemographic Variables

A significant difference in attitudes toward HBV‐infected patients was observed among college, high school, and university students (Table 4). Specifically, 47.37% of college students reported feeling uncomfortable sitting next to an HBV‐infected person, compared to 26.48% of high school students and 27.65% of university students (*p* = 0.001).

**Table 4:** Sociodemographic and attitude‐related characteristics about HBV and its vaccination of the study sample.

Characteristics	Gender			Marital status			Educational status			
	Male *n* (%)	Female *n* (%)	*p*	Married *n* (%)	Unmarried *n* (%)	*p*	High school *n* (%)	College *n* (%)	University *n* (%)	*p*
Do you feel uncomfortable sitting with a HBV‐infected person?	Total (*n* = 700)									
Yes	95 (31.99)	111 (27.54)	0.209	15 (34.88)	191 (29.07)	0.258	58 (26.48)	36 (47.37)	112 (27.65)	**0.001**
No	202 (68.01)	292 (72.46)		28 (65.12)	466 (70.93)		161 (73.52)	40 (52.63)	293 (72.35)	
You do not mind shaking hands/hugging with a HBV‐infected person.	Total (*n* = 700)									
Yes	88 (29.63)	130 (32.26)	0.509	18 (41.86)	200 (30.44)	0.083	55 (25.11)	35 (46.05)	128 (31.6)	**0.003**
No	209 (70.37)	273 (67.74)		25 (58.14)	457 (69.56)		164 (74.89)	41 (53.95)	277 (68.4)	
Do you help your family member to take vaccine against HBV?	Total (*n* = 700)									
Yes	292 (98.32)	398 (98.76)	0.428	42 (97.67)	648 (98.63)	0.472	214 (97.72)	75 (98.68)	401 (99.01)	0.199
No	5 (1.68)	5 (1.24)		1 (2.33)	9 (1.37)		5 (2.28)	1 (1.32)	4 (0.99)	
Do you suggest your friends, relatives, or other persons to take vaccine against HBV?	Total (*n* = 700)									
Yes	292 (98.32)	397 (98.51)	0.534	43 (100)	646 (98.33)	0.495	214 (97.72)	75 (98.68)	400 (98.77)	0.327
No	5 (1.68)	6 (1.49)		0	11 (1.67)		5 (2.28)	1 (1.32)	5 (1.23)	

*Note:* Bold values indicate statistically significant.

However, when asked if they would mind shaking hands or giving hugs to an HBV‐infected person, 46.05% of college students responded that they did not mind, in contrast to 25.11% of high school students and 31.6% of university students (*p* = 0.003).

No statistically significant differences were observed between male and female students regarding discomfort sitting next to an HBV‐positive individual. Additionally, 29.63% of male and 41.86% of married students indicated that they did not mind shaking hands or giving hugs to an HBV‐positive person, compared to 32.26% of female and 30.44% of unmarried students (*p* = 0.509 and *p* = 0.083, respectively). In contrast, no significant association was found between attitudes toward HBV vaccination and sociodemographic variables such as gender, marital status, and educational status (Table 4). Most participants expressed a willingness to support family members and encourage friends or others to receive the HBV vaccination.

### 3.4 Correlation Between HBV Vaccination Status and Sociodemographic Variables of the Study Sample

The study found a strong correlation between gender and vaccination status, with 58.88% of male participants receiving the HBV vaccine, compared to 43.1% of female participants (OR = 1.435, 95% CI = 1.253–1.873, *p* = 0.027). A significantly lower vaccination rate was also observed among university students (42.11%) compared to college (44.44%) and high school (86.3%) students (OR = 1.917, 95% CI = 0.752–17.869, *p* = 0.001). Furthermore, a higher vaccination rate was demonstrated among married students (44.19%) compared to unmarried students (34.25%) (OR = 1.701, 95% CI = 1.345–1.753, *p* = 0.124) (Table 5).

**Table 5:** Correlation between HBV vaccination status and sociodemographic variables of the study sample.

Predictors	Vaccination status		*χ* ^2^ test value	*p*	Odds ratio (95% confidence interval)
	Yes	No			
Gender					
Male	264 (41.12)	378 (58.88)	1.439	**0.027**	1.435 (1.253–1.873)
Female	33 (56.9)	25 (43.1)			
Marital status					
Married	19 (44.19)	24 (55.81)	1.756	0.124	1.701 (1.345–1.753)
Unmarried	225 (34.25)	432 (65.75)			
Educational status					
High school	189 (86.3)	30 (13.7)	62.838	**0.001**	1.917 (0.752–17.869)
College	180 (44.44)	225 (55.56)			
University	32 (42.11)	44 (57.89)			

*Note:* Bold values indicate statistically significant.

## 4 Discussion

The prevalence of HBV infection among various population groups in Bangladesh has fluctuated over the past three decades, with the majority of cases being asymptomatic [19]. This indicates the persistence of the source of infection in the community [20]. Surveys of infectious diseases are essential tools for identifying problems, offering fixes, and implementing policies [21]. Students play a crucial role in preventing infectious diseases within their communities, and therefore, the present study aimed to assess the knowledge and attitudes toward HBV and its vaccination among high school, college, and university students of the Rajshahi division of Bangladesh.

The present study showed that regarding the knowledge of HBV and its transmission, university students had better knowledge than high school students (*p* > 0.05). High school students demonstrated more positive attitudes toward HBV‐infected individuals compared to university students. Moreover, a significantly lower rate of vaccination status was observed among participants from the university (44.44%) than those from college (42.11%) and high school (86.3%) [odds ratio (OR) = 1.917, 95% CI = (0.752–17.869), *p* = 0.001]. Compared to female students (43.1%), a greater percentage of male students (58.88%) did not receive a vaccination [OR = 1.435, 95% CI = (1.253–1.873), *p* = 0.027].

According to our study, high school students lack basic information about the many elements of HBV infection, including the routes of transmission, pathogenesis, and prevention. In contrast to these findings, it was found that university students were more knowledgeable about the various modes of spread of infection and believed that the HBV vaccine prevented it. It was expected that the knowledge level will upgrade by year and accordingly, the level of knowledge is higher for the higher levels compared to the junior level. Our results support this conception and another study [22], but are in contrast with the study of dental students in Saudi Arabia [23].

A notable finding was the prevalence of misconceptions about HBV transmission. Specifically, a significant proportion of high school students (50.46%) incorrectly believed that HBV can be spread through kissing. Additionally, while a majority of university students (56.79%) correctly identified HBV as a sexually transmitted disease, a substantial percentage of college and high school students demonstrated a lack of awareness in this area. These results align with previous studies, such as that by Aslam et al. [24], which reported similar misperceptions among medical students. This highlights the need for targeted educational interventions to address specific knowledge gaps regarding HBV transmission. The exact knowledge of students about the transmission methods of HBV forces them to take precautions in their daily lives and raise awareness among their families and the broader community. Educational programs, health campaigns, and healthcare initiatives may contribute to improving knowledge of HBV infection.

Contrary to expectations, high school students demonstrated more positive attitudes toward HBV‐infected individuals compared to university students. Moreover, contrary to previous studies, more positive attitudes were observed in high school students than in college and university students [22, 25, 26, 27]. This may be due to the reason that their positive attitudes have potentially influenced by school‐based health campaigns, teacher/parent guidance, or peer networks. Conversely, compared to high school and college students, a greater percentage of university students reported having good attitudes toward recommending their relatives, family members, or others to take the vaccine.

Furthermore, it was demonstrated that 86.3% of high school students, 44.44% of college students, and 42.11% of university students received the HBV vaccine. Owing to their exclusion from the HBV EPI program, college and university students had a lower vaccination rate. A significant regional variation in low vaccination rate (41% among medical students in Bangladesh; *p* < 0.01) was observed in a previous study [16]. Lack of awareness, concerns about side effects, perceived low risk or necessity, and lack of motivation were reported for this low vaccination rate [16]. Our study supports this study that low vaccination coverage was demonstrated due to lack of proper knowledge and awareness.

Students in high school, college, and university in Bangladesh are between the ages of 11 and 16, 16 and 19, and 19 and 25 years, respectively, in accordance with the country's academic policies. Although our survey was conducted in 2021 and Bangladesh's HBV vaccination program began in 2005, college and university students were not included in this program. Moreover, some nongovernmental agencies offered a safe and effective HBV vaccine [28] for those who were not included in the immunization program. However, a low vaccination rate indicates a lack of commitment, understanding, and knowledge to prevent HBV infection.

Similar to previous study [29], the current study showed distinct differences in terms of gender and marital status among students. However, in contrast to another study where gender and knowledge were not significantly correlated, our study showed a significant relationship between gender and knowledge [30].

Bangladesh, a developing nation, ranks 142nd out of 187 countries on the UN Human Development Index, indicating less than excellent health indices. As more than one‐third of the population lives below the poverty line and has a precarious health status, many patients are unable to afford the expensive treatment of diseases caused by HBV infection. Therefore, prevention is the sole defense mechanism against viral hepatitis.

Over one‐third of the total population lives below the poverty line and has a fragile health structure; many patients cannot pay for costly treatment of the diseases caused by HBV infection. Hence, prevention is the only safeguard against viral hepatitis [31].

The primary strength of this study is that it was conducted among school, college, and university students in the Rajshahi Division of Bangladesh for the first time. Another strength of this study was its large sample size, which was used to compute the knowledge and attitudes toward HBV and its vaccination status, thus reducing the possibility of bias. However, there are several drawbacks. The main drawback is that the vaccination status was self‐reported and not verified by measuring the antibody titer. Therefore, the conclusions of this study may have been affected by recollection bias and inaccurate information. Additionally, the survey was conducted at different high schools, colleges, and universities in the Rajshahi Division of Bangladesh which may not represent the actual situation of the country. As vaccination status was self‐reported, the possibility of recall bias, social desirability bias, and misclassification must be acknowledged. Participants may have overreported vaccination due to perceived social expectations or underreported due to forgetfulness. The absence of serological testing to confirm immunity further limits the precision of the vaccination coverage estimates. However, this will provide background information for future research in the country. Moreover, a more thorough analysis using both qualitative and quantitative methods from various socioeconomic backgrounds is needed to interpret the original knowledge, attitudes, and practices of HBV vaccination status of the country and to increase the awareness of HBV vaccination.

## 5 Conclusion

Knowing facts and having proper awareness of HBV and its vaccination are critical to prevent the spread of this infection. The findings of this study support the need for health education and immunization campaigns among school, college, and university students to prevent HBV infections.

## Author Contributions

**Mst. Hajera Khatun:** conceptualization, supervision, writing – original draft, formal analysis, methodology. **Md. Arif Hossain:** investigation, writing – original draft, writing – review and editing. **Jaytirmoy Barmon:** supervision, formal analysis, writing – review and editing. **Mst. Sharmin Khatun:** investigation, writing – review and editing. **Md. Milton Hossain:** investigation, writing – review and editing. **Md. Minhazul Islam:** investigation, writing – review and editing. **Al Mamun:** writing – review and editing. **Md. Ajijur Rahman:** formal analysis, writing – review and editing, methodology. All authors have read and approved the published version of the manuscript. Mst. Hajera Khatun had full access to all of the data in this study and takes complete responsibility for the integrity of the data and the accuracy of the data analysis.

## Disclosure

The lead author Mst. Hajera Khatun affirms that this manuscript is an honest, accurate, and transparent account of the study being reported; that no important aspects of the study have been omitted; and that any discrepancies from the study as planned (and, if relevant, registered) have been explained.

## Ethics Statement

This study was approved by the Ethical Review Committee of Varendra University (Ref: VU/ERC/2023/005).

## Consent

Written consent forms were provided to all participants. Data were collected from participants who provided consent.

## Conflicts of Interest

The authors declare no conflicts of interest.

## Data Availability

The data used and/or analyzed in the current study are available from the corresponding author upon valid requests.
